# Radiographic Knee Osteoarthritis and Knee Pain: Cross-sectional study from Five Different Racial/Ethnic Populations

**DOI:** 10.1038/s41598-018-19470-3

**Published:** 2018-01-22

**Authors:** Ke Wang, Hyun A. Kim, David T. Felson, Ling Xu, Dong H. Kim, Michael C. Nevitt, Noriko Yoshimura, Hiroshi Kawaguchi, Jianhao Lin, Xiaozheng Kang, Yuqing Zhang

**Affiliations:** 1 Clinical Epidemiology Research & Training Unit, Boston University School of Medicine, Boston, MA United States; 2 Department of Medicine, Hallym University, Chunchun, South Korea; 3 Department of Obstetrics and Gynecology, Peking Union Medical College, Beijing, China; 4 Hallym Research Institute of Clinical Epidemiology, Hallym University, Chunchun, South Korea; 5 Department of Epidemiology, University of California, San Francisco, CA United States; 6 Department of Joint Disease Research, The University of Tokyo, Tokyo, Japan; 7 Department of Orthopedic Surgery, People’s Hospital, Peking University, Beijing, China; 8 The Department of Thoracic Surgery, Beijing Cancer Hospital and Institute, Beijing, China; 9 Present Address: JCHO Tokyo Shinjuku Medical Center, Tokyo, Japan

## Abstract

The weak correlation between pain and structural changes in knee osteoarthritis is widely reported. In a previous within-person, knee-matched case-control study among Caucasians, the severity of radiographic osteoarthritis (ROA) was strongly associated with both the presence of frequent knee pain and pain severity. We studied the association between ROA and knee pain in five racial/ethnic populations by using the same method. Subjects were selected from China; Japan; Korea and the United States. Among subjects with knees discordant for either frequent knee pain or pain severity, we examined the relationship between ROA and the presence of frequent knee pain using conditional logistic regression, and between ROA and pain severity using a stratified proportional odds model with an amalgamating conditional likelihood. In total, 252 urban Chinese, 221 rural Chinese, 297 Japanese, 122 Korean, 1,735 Caucasian, and 394 African-American patients were included. There was a strong dose-response relationship between the severity of ROA and the prevalence of frequent knee pain in all five racial/ethnic populations. Even mild ROA was significantly associated with frequent knee pain. In addition, ROA was also strongly associated with the severity of knee pain. These results show that structural pathology is associated with knee pain in different ethnic populations.

## Introduction

Osteoarthritis (OA) is the most common joint disorder and the leading cause of disability among the elderly^1,2^. Symptomatic knee OA occurs in approximately 12% of people aged 60 years or older^3^. The number of individuals with clinical OA in the United States increased from 21 million in 1995 to nearly 27 million by 2005^3^.

Pain from knee OA is a key symptom informing the decision to seek medical care, and an important antecedent to disability^4^. Furthermore, symptomatic knee OA is the most common reason for total knee replacement, and the use of this surgical procedure has increased rapidly over the past few decades. For example, the number of total knee replacements increased by 69%, from 328,800 in 1997 to 555,800 in 2005, with a cost exceeding $11 billion^5^. In South Korea, the number of total knee replacements increased almost two-fold between 2002 and 2005^6^. Such a rapid increase in the prevalence of this already common disease suggests that OA will have a growing impact on healthcare and public health systems in the future^3^.

Radiographs have long been considered as the reference standard for the assessment of OA, and the Kellgren and Lawrence (K/L) radiographic grading scheme and atlas have been in use for more than four decades. To date, the majority of studies have reported that radiographic OA (ROA) is poorly correlated with knee symptoms, and most risk factors for ROA are not strong predictors of knee pain^1,7^. Considering the traditional concept that nociceptive sensory input from tissue damage is the main mechanism leading to pain, the weak correlation between pain and knee structural changes is somewhat counter-intuitive. Pain perception is complex, however, and knee pain is frequently associated with non-OA variables, such as psychosocial factors, education, economic statusas well as local pathology^8^. Previously, a within-person, knee-matched case-control study included patients with knees discordant for the presence of pain or pain severity; in that study, Neogi and colleagues demonstrated that the severity of radiographic knee OA was strongly associated with both the presence of frequent knee pain and pain severity^9^. The study also showed that even among knees with mild ROA (K/L grade = 2), there was an increase in the frequency of knee pain compared to those without ROA (K/L grade = 0 or 1). These findings indicate that the pathological changes revealed by radiographs are indeed correlated with pain.

Neogi *et al*. demonstrated a strong relationship between X-ray findings and pain in two cohorts from the United States, but both cohorts comprised predominantly Caucasian subjects. To date, there remains a paucity of information as to whether the relationship between radiographic knee OA and knee pain is consistent across racial groups or geographic regions. In multiple studies, the tolerance to, perception of, and response to experimental and acute pain have been reported to vary according to ethnicity and culture^10,11^. For example, the cold pressor test, which elicits an emotional/motivational pain experience on immersion of a limb in cold water, revealed an ethnic difference in terms of pain tolerance^12^.Thus, the question of whether findings obtained in Caucasians can be generalised to other racial or ethnic populations remains uncertain. We assembled data from five OA studies, conducted in China, Japan, South Korea, and the United States, and examined whether radiographic knee OA was associated with the presence of frequent knee pain and pain severity in five racial/ethnic populations using a within-person, knee-matched case-control design.

## Results

This study included 3,021 individuals who had unilateral frequent knee pain or knees that were discordant for pain severity. Of them, 252 were urban Chinese (Beijing Osteoarthritis Study, BOA), 221 were rural Chinese (Wuchuan Osteoarthritis Study, WOA), 297 were Japanese (Research on Osteoarthritis/osteoporosis Against Disability Study, ROAD), 122 were Korean (Hallym Aging Study, HAS), 1,735 were Caucasian (Osteoarthritis Initiative, OAI), and 394 were African-American (OAI).

The demographic and anthropometric characteristics of the participants in each study are presented in Table 1. The age of the study participants ranged from 32–95 years, and slightly more than 60% of the participants were women. Participants from the three Asian countries were lighter, shorter, and had a lower mean body mass index than their counterparts in the United States. The majority of Caucasians (98%) and African-Americans (90%) had completed a high school education. On the other hand, approximately 55% of the Japanese, 77% of the Korean, and 85% of the Chinese participants in the Beijing study completed only 9 years (or less) of schooling. More than 67% of the Chinese participants in the WOA study did not receive any formal education.

**Table 1:** Distribution of demographic and anthropometric factors among the participants of the five studies.

Characteristics	BOA	WOA	HAS	ROAD	OAI White	OAI Black
	(N = 252)	(N = 221)	(N = 122)	(N = 297)	(N = 1,735)	(N = 394)
Age (years)^a^	68 (6)	57 (8)	70 (8)	71 (9)	61 (9)	62 (9)
Sex (male, %)	29	45	42	29	45	30
Height (cm)^a^	158 (8)	160 (9)	156 (9)	153 (9)	169 (93)	168 (86)
Weight (kg)^a^	66 (12)	58 (10)	61 (10)	56 (10)	81 (16)	86 (15)
BMI (kg/m^2^)^a^	26 (4)	23 (3)	25 (3)	24 (3)	28 (5)	31 (5)

^a^Mean (SD).

Abbreviations: BOA, Beijing Osteoarthritis Study, China; WOA, Wuchuan Osteoarthritis Study, China; ROAD, Research on Osteoarthritis/osteoporosis Against Disability Study, Japan; HAS, Hallym Aging Study, South Korea; OAI, Osteoarthritis Initiative, United States; BMI, body mass index.

As shown in Table 2, there was a strong dose-response relationship between the severity of knee ROA and the prevalence of frequent knee pain in all five racial/ethnic populations (p for trend < 0.01 for all studies). Even mild ROA (K/L grade = 2) was significantly associated with frequent knee pain. Compared to knees without evidence of ROA (K/L = 0), the odds ratios of frequent knee pain among knees with mild ROA were 6.8 (95% confidence interval (CI): 2.6–17.8) among urban Chinese, 3.8 (95% CI: 1.1–12.9) among rural Chinese, 3.0 (95% CI: 0.8–11.6) among Japanese, 6.6 (95% CI: 1.7–26.2) among Koreans, 3.7 (95% CI: 2.6–5.1) among Caucasians, and 3.5 (95% CI: 1.7–7.3) among African-Americans.

**Table 2:** Association between radiographic knee OA and the presence of frequent knee pain in five racial/ethnic populations^a^.

Racial/ethnic origin (study)	Frequent knee pain	Kellgren and Lawrence grade			
		0	1	2	3–4
Urban Chinese	No	141 (56.0)	48 (19.0)	38 (15.1)	25 (10.0)
(BOA)	Yes	113 (44.8)	54 (21.4)	38 (15.1)	47 (18.7)
	OR (95% CI)	1	1.9 (0.9, 3.7)	6.8 (2.6, 17.8)	16.8 (5.3, 53.2)
Rural Chinese	No	194 (87.8)	9 (4.1)	14 (6.3)	4 (1.8)
(WOA)	Yes	180 (81.4)	12 (5.4)	22 (10.0)	7 (3.2)
	OR (95% CI)	1	2.1 (0.9, 5.0)	3.8 (1.1, 12.9)	9.4 (1.7, 52.5)
Japanese	No	27 (9.1)	87 (29.3)	126 (42.4)	57 (19.2)
(ROAD)	Yes	23 (7.7)	62 (20.9)	111 (37.4)	101 (34.0)
	OR (95% CI)	1	1.1 (0.3, 3.9)	3 (0.8, 11.6)	24.2 (4.8, 121.8)
Koreans	No	32 (32.7)	41 (41.8)	20 (20.4)	5 (5.1)
(HAS)	Yes	25 (25.5)	37 (37.8)	28 (28.6)	8 (8.2)
	OR (95% CI)	1	2.3 (0.9, 6.0)	6.6 (1.7, 26.2)	10 (2.0, 49.9)
African-Americans	No	75 (35.4)	36 (17.0)	73 (34.4)	28 (13.2)
(OAI)	Yes	57 (26.9)	28 (13.2)	75 (35.4)	52 (24.5)
	OR (95% CI)	1	1.7 (0.9, 3.3)	3.5 (1.7, 7.3)	8.5 (3.6, 20.3)
Caucasians	No	358 (42.9)	197 (23.6)	179 (21.4)	101 (12.1)
(OAI)	Yes	249 (29.8)	142 (17.0)	183 (21.9)	261 (31.3)
	OR (95% CI)	1	1.8 (1.3, 2.4)	3.7 (2.6, 5.1)	15.8 (10.1, 24.8)

N(%), Abbreviations: CI, Confidence interval.

Conditional logistic regression results for subjects with knees discordant for the presence of frequent knee pain.

Subjects included in Table 2 with bilateral knee pain of discrepant severity (in the HAS and OAI studies) were excluded.

Abbreviations: BOA, Beijing Osteoarthritis Study, China; WOA, Wuchuan Osteoarthritis Study, China; ROAD, Research on Osteoarthritis/osteoporosis Against Disability Study, Japan; HAS, Hallym Aging Study, South Korea; OAI, Osteoarthritis Initiative, United States; OR, odds ratio; CI, confidence interval.

**Figure 1:**
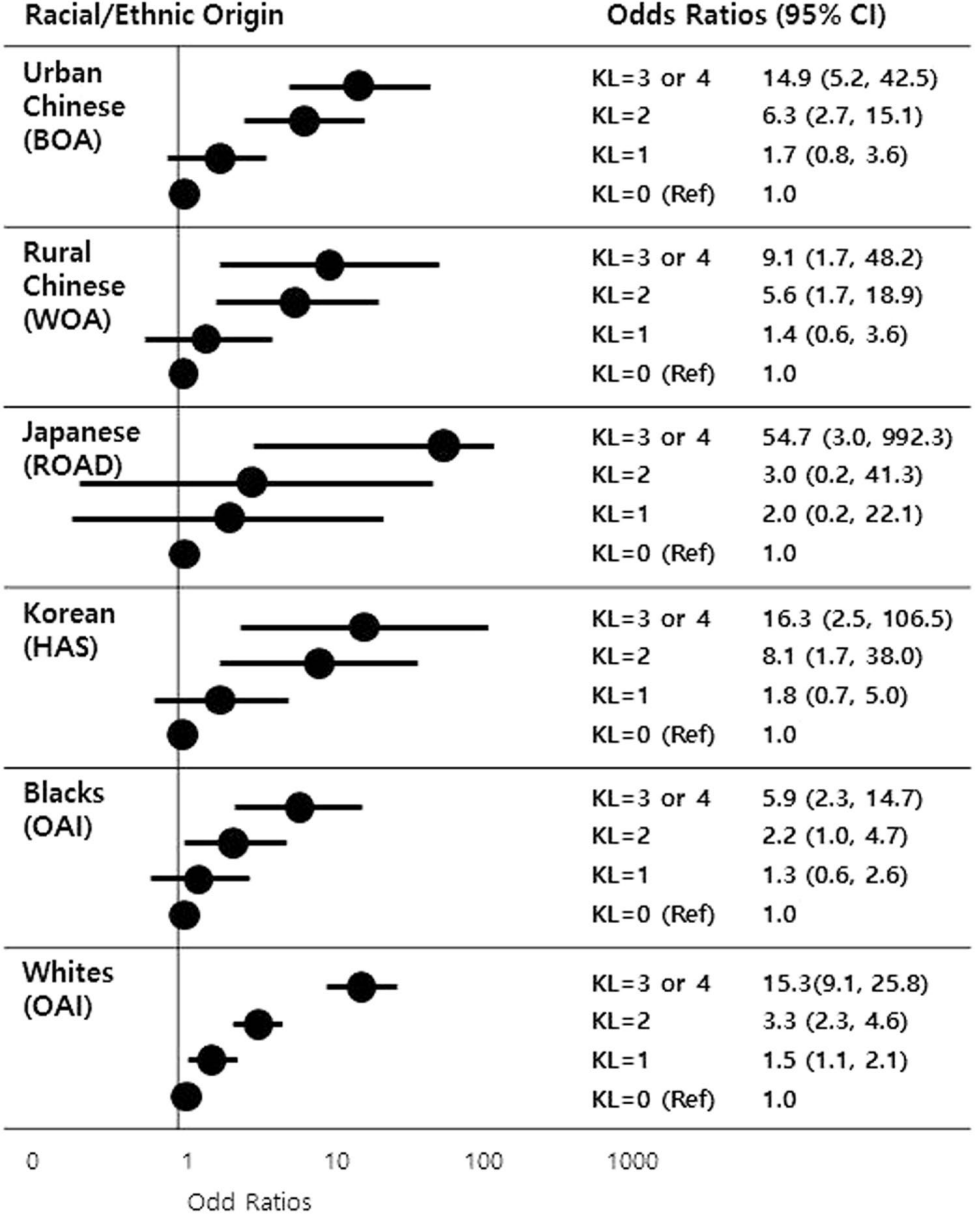
Association between radiographic knee OA and knee pain severity in five racial/ethnic populations*. *Amalgamating conditional logistic regression results for subjects with knees discordant for the severity of pain. Abbreviations: BOA, Beijing Osteoarthritis Study, China; WOA, Wuchuan Osteoarthritis Study, China; ROAD, Research on Osteoarthritis/osteoporosis Against Disability Study, Japan; HAS, Hallym Aging Study, South Korea; OAI, Osteoarthritis Initiative, United States; CI, confidence interval; K/L, Kellgren &and Lawrence grade.

Figure 1 depicts the association of K/L score with knee pain severity. In all five racial/ethnic populations, the severity of ROA, indicated by K/L grade, and the severity of knee pain were positively associated (p for trend < 0.001 for all studies). Compared to knees with no ROA (K/L grade = 0), the odds ratios of the degree of pain severity for knees with mild ROA (K/L grade = 2) were 6.3 (95% CI: 2.7–15.1) among urban Chinese, 5.6 (95% CI: 1.7–18.9) among rural Chinese, 3.0 (95% CI: 0.2–41.3) among Japanese, 8.1 (95% CI: 1.7–38.0) among Koreans, 2.2 (95% CI: 1.0–4.7) among African-Americans, and 3.3 (95% CI: 2.3–4.6) among Caucasians.

## Discussion

Using data collected from five racial/ethnic populations, we showed that radiographic knee OA was strongly associated with the presence and severity of knee pain. Such an association was found even in knees with mild disease. Our findings, in addition to the results from a previous study^9^, demonstrate that structural lesions, defined by the K/L score on radiographs, are indeed strongly correlated with one of the major clinical symptoms of OA, i.e. knee pain.

A recent meta-analysis showed that ROA of the knee at baseline was inconsistently associated with worsening knee pain and did not predict physical functioning^13^. Unlike structural lesions in OA, pain is a subjective experience unique to each individual, with natural variability among individuals in terms of sensitivity to, and perception and tolerance of, pain stimuli. A number of factors (e.g. genetic predisposition^14,15^, prior experience^16^, idiosyncratic appraisals^17^, expectations^18^, current mood^19^, and the socio-cultural environment^20–22^) influence an individual’s response to painful stimuli. In addition, central sensitisation, as measured by temporal summation or neuroimaging, was shown to be significantly associated with knee OA symptom severity, while there was no association between sensitisation and radiographic severity^23^. Unless all of these risk factors are measured and controlled, studies comparing groups of individuals with respect to the effect of pathological lesions on the risk or severity of knee pain are susceptible to residual confounding bias^24^.

A within-person, knee-matched case-control study design^9^ ensures that all person-level factors that are associated with knee pain are distributed evenly between both knees, eliminating their confounding effects between comparison groups. In contrast to previous findings of there being only a modest association between ROA and knee pain, the authors found that knees with K/L grades of 1, 2, 3, and 4 had 1.2 (95% CI: 0.6–2.5), 3.1 (95% CI: 1.5–6.5), 15.1 (95% CI: 5.6–41.2), and 73 (95% CI: 16.2–331) times higher odds of frequent knee pain, respectively, compared to knees with a K/L grade of 0 in the Framingham OA Study, with a similar trend for the Multicenter OA Study.

Several aspects of this study are notable. First, we assembled data from five studies that were conducted in four countries and included different five racial/ethnic populations. The participants in these studies had different socio-cultural and educational backgrounds and anthropometrical characteristics, engaged in different recreational and occupational activities, and showed differences in the prevalence of knee OA. Compared with other countries, especially large difference in economic and cultural lifestyle exists in China, considering the dimension of its area and historic perspectives. For example, in a population-based cohort study conducted among residents living in rural areas in Wuchuan, China, while the overall prevalence of radiographic knee OA among rural residents was similar to that among urban residents in Beijing, the symptomatic OA was twice as prevalent in Wuchuan^25^ as that in Beijing^26^. However, in all of the studies, the results demonstrated that radiographic knee OA, even mild OA, is associated with knee pain and pain severity; which increases the validity of the current findings. Second, although the sample size varied markedly in the five studies, resulting in wider confidence intervals for some effect estimates (e.g. Fig. 1, ROAD), the consistency of the overall trend across the cohorts still indicates a positive association between ROA and knee pain severity. Finally, while the questions used to assess knee pain and pain severity varied among the five included studies, the findings themselves did not appear to be influenced by this potential limitation. This again indicates that structural lesions do indeed contribute to knee pain, regardless of how they are assessed.

Our study had several limitations. First, due to the study design, the analyses used to assess the association between radiographic features and the presence of frequent knee pain were restricted mostly to individuals with unilateral knee pain. As a result, the conclusions of our study may not generalise to individuals with bilateral knee pain. Nevertheless, it is difficult to imagine that the association between structural lesions revealed by radiographs and knee pain observed among individuals with unilateral knee pain would not apply to those with bilateral knee pain. Second, our study looked at the tibiofemoral joint only, but it is possible that some of the knee pain could have arisen from pathology in the patellofemoral joint. The within-person, knee-matched approach may have an inherent limitation due to selectively favouring knee OA with a traumatic aetiology. Lastly, our data show that some cases of knee pain still cannot be explained by reference to the K/L grade, since 43.9% of our subjects with a K/L grade of 0 had knee pain, while 31.6% of those with a K/L grade of 3–4 did not.

In conclusion, we confirmed the finding of a previous study that radiographic knee OA is strongly associated with the presence and severity of knee pain, in this case in five different racial/ethnic populations. Differences in musculoskeletal pain perception have been reported according to race and ethnicity; however, difference regarding the structure-symptom relationship in knee OA has not previously been reported. These results show that structural pathology captured by radiographic imaging, such as osteophytes and joint space narrowing, are associated with knee pain in different ethnic populations.

## Methods

### Study Cohorts

The data used for the current analysis were extracted from five study cohorts: the Beijing Osteoarthritis Study(BOA), China, the Wuchuan Osteoarthritis Study(WOA), China, the Research on Osteoarthritis/osteoporosis Against Disability(ROAD) Study, Japan, the Hallym Aging Study(HAS), South Korea, and the Osteoarthritis Initiative(OAI), United States. The Ethics Committee of each participating centre approved the study protocol(the Peking University Health Science Center Ethics Committee for BOA and WOA, the ethics committees of the University of Tokyo and the Tokyo Metropolitan Geriatric Medical Center for ROAD study, The ethics committee of Hallym University for HAS study), and written informed consent was obtained from all study participants. Osteoarthritis Initiative database is available for public access at http://www.oai.ucsf.edu/, thus additional ethics committee approval was not obtained. All methods were performed in accordance with the relevant guidelines and regulations in each participating centers. The details of each study have been published previously, and we briefly describe each of the studies in the following paragraphs.

### The Beijing Osteoarthritis Study (BOA)

A random sample of residents aged ≥60 years was recruited from four central districts of Beijing (an urban area), China to study OA in major joints, including the knee, and their risk factors. Participants completed a home interview that included questions regarding knee pain and its severity. Bilateral, anteroposterior (AP) fully extended weight-bearing knee radiographs were taken for all participants in the hospital according to the Framingham OA Study protocol. Tibiofemoral radiographs were evaluated according to K/L grade. One bone and joint radiologist read knee films according to the reading protocols of the Framingham OA Study. The weighted kappa on K/L grade was 0.79 (95% confidence interval 0.73–0.84) for intra-reader reliability. The small number of disagreements did not occur in any particular direction, suggesting that there was no likelihood of bias in estimates. Data on both knee pain and knee ROA were available from 2,513 participants^26^.

### The Wuchuan Osteoarthritis Study (WOA)

A random sample of residents aged ≥50 years was recruited from Wuchuan county (a rural area), Inner Mongolia, China to study the prevalence of knee OA and its risk factors. Participants completed a home interview that included questions regarding knee pain and its severity. Bilateral, AP fully extended weight-bearing knee radiographs were taken for all participants in the hospital according to the Framingham OA Study protocol. Radiographs were evaluated according to K/L grade. An investigator from Wuchuan Osteoarthritis Study was trained at Boston University. The weighted kappa on KL grade for the intra-rater reliability was 0.92 (95% CI: 0.86–0.99). Data on both knee pain and knee ROA were available from 1,025 participants^27^.

### The Research on OA/osteoporosis Against Disability Study (ROAD)

Study participants were randomly recruited from Itabashi, Hidakagawa, and Taiji, Japan to determine the environmental and genetic background of bone and joint diseases^28^. Participants, aged ≥60 years (Itabashi) or ≥40 years (Hidakagawa and Taiji) completed an interviewer-administered questionnaire that included items regarding knee pain. Bilateral AP weight-bearing knee radiographs with foot map positioning and fluoroscopic guidance were taken and ROA was evaluated using the K/L grade. Knee radiographs were read without knowledge of participant clinical status by a single well-experienced orthopaedist. One hundred other radiographs were also scored by two experienced orthopeadic surgeons using the same atlas for inter-rater variability. The intra- and inter- reader variabilities evaluated for KL grade (0–4) were confirmed by the kappa analysis to be sufficient for assessment (0.86 and 0.80, respectively). Data on both the presence of frequent knee pain and knee ROA were available from 2,981 subjects.

### The Hallym Aging Study (HAS)

A random sample of residents was recruited from Chuncheon, Korea to investigate quality of life and health. Approximately 70% of the participants were aged ≥65 years. Subjects completed a face-to-face interview and underwent bilateral, AP semi-flexed weight-bearing knee radiography that involved the use of a Plexiglas frame (SynaFlexer; Synarc, San Francisco, CA, USA) to standardise knee positions. Knee ROA was evaluated according to K/L grade. Radiographs were read twice by one reader, an academically based rheumatologist. Films allocated different K-L grades at the two readings were adjudicated by consensus between the original reader and a second reader, another academically based rheumatologist. The reproducibility of intra-reader assessments was high (for OA vs no OA, κ = 0.89). Data on both knee pain and knee ROA were available from 494 individuals^29^.

### OA Initiative (OAI)

The OAI study is a multi-centre longitudinal observational study focusing primarily on knee OA. Individuals aged between 45–79 years were recruited at four centres across the United States to study the natural history of, and risk factors for, the onset and progression of knee OA(OAI – https://www.oai.ucsf.edu/). At baseline and yearly follow-up clinic visits, data on clinical parameters (e.g. presence of frequent knee pain and pain severity) and radiographic imaging of the knee (e.g. bilateral, AP fixed flexion weight-bearing knee radiographs) were collected. Radiographic knee OA was assessed according to K/L grade. Radiographs were read independently by two study readers, a musculoskeletal radiologist and a rheumatologist at Boston University. In the case of discrepancy, readings were adjudicated by consensus with a third reader. Of the 4,796 subjects included in the OAI, 3,496 Caucasians and 731 African-Americans had both knee pain and knee radiograph data.

### Knee Pain Assessment

As shown in Table 3, the questions used to assess the presence of frequent knee pain and pain severity varied among the studies. All subjects were asked about the presence of frequent pain in each of their knees. Participants in the HAS and OAI studies were asked to rate the pain severity for each knee separately. However, in the BOA and WOA studies, the question regarding knee pain severity was only presented to subjects who responded positively to the frequent knee pain question, and the question on pain severity was not knee-specific. In the ROAD study, data on knee pain severity were only collected at the person, rather than the knee, level. Thus, for the BOA, WOA, and ROAD studies, we limited our analysis to subjects who had reported unilateral frequent knee pain and assumed that pain severity referred to the knee that experienced frequent knee pain. Knee symptoms were rated by assessors blinded to the radiographic findings.

**Table 3:** Questions used to assess frequent knee pain and knee pain severity in the five studies.

Study	Assessment of frequent knee pain	Assessment of knee pain severity
BOA	In the past 12 months, have you had pain, aching, or stiffness lasting at least a month in your knee?	How severe was the pain usually? (person-specific)
	(1) Yes	(1) Usually bearable
	(2) No	(2) Sometimes unbearable
		(3) Mostly or always unbearable
WOA	In the past 12 months, have you had pain, aching, or stiffness lasting most days of at least one month in your knee?	How severe was the pain usually? (person-specific)
	(1) Yes	(1) Usually bearable
	(2) No	(2) Sometimes unbearable
		(3) Mostly or always unbearable
ROAD	In the past 12 months, have you had pain, aching, or stiffness lasting most days of at least one month in your knee?	WOMAC pain score (person-specific)
	(1)Yes	
	(2) No	
HAS	Have you had pain, aching, or stiffness lasting at least one month in your knee?	Visual analogue scale (knee-specific)
	(1) Yes	
	(2) No	
OAI	Have you had knee pain, aching or stiffness for more than half the days of one month in the past 12 months?	WOMAC pain score (knee-specific)
	(1) Yes	
	(2) No	

Abbreviations: BOA, Beijing Osteoarthritis Study, China; WOA, Wuchuan Osteoarthritis Study, China; ROAD, Research on Osteoarthritis/osteoporosis Against Disability Study, Japan; HAS, Hallym Aging Study, South Korea; OAI, Osteoarthritis Initiative, United States; WOMAC, Western Ontario and McMaster Universities Arthritis Index.

The English in this document has been checked by at least two professional editors, both native speakers of English. For a certificate, please see: http://www.textcheck.com/certificate/TpMQtO.

### Radiographic Assessment

We considered a knee to have tibiofemoral ROA if its K/L grade was ≥2. In addition, we further classified a knee as having mild ROA if the K/L score was 2, and moderate-to-severe ROA if the K/L score was ≥3. Radiographs from all studies were scored by assessors blinded to symptom status.

### Statistical Analyses

We conducted a within-person, knee-matched case-control study to examine the relationships between the severity of ROA and the presence of frequent knee pain and pain severity. To examine the relationship between knee ROA and the prevalence of frequent knee pain, we identified individuals who indicated that they experienced unilateral frequent knee pain. The knee with frequent knee pain served as the case knee, and the contralateral knee without frequent pain served as the control; thus, the two knees of each individual formed a matched pair. We assessed the association between ROA and the presence of frequent knee pain using conditional logistic regression.

To evaluate the relationship between knee ROA and the severity of knee pain, we grouped pain severity into three categories: *no pain*, *usually bearable*, and *sometimes/mostly/always unbearable* for the BOA and WOA; 0, 1–70, and 71–100 on visual analogue scales for the HAS; and 0, 1–3, and 4+ on Western Ontario and McMaster Universities Arthritis Index pain score for the ROAD and OAI. We identified subjects in whom knees were discordant for knee pain severity. As noted above, for the BOA, WOA, and ROAD studies, knee pain severity was assessed on a person level rather than on a knee level; thus, we limited our analysis to subjects who had reported unilateral frequent knee pain. We performed amalgamating conditional logistic regression analysis to examine the association between ROA and knee pain severity^30^, which is an ordinal outcome variable. As an extension to conditional logistic regression, amalgamating conditional logistic regression analyzes matched ordinal data. All statistical analyses were performed using SAS software (ver. 9.1; SAS Institute, Cary, NC, USA), except for the amalgamating logistic regression, which was performed in R software (ver. 2.8.1.; R Development Core Team, Vienna, Austria).
